# Racial and Ethnic Disparities in Adult Vaccination: A Review of the State of Evidence

**DOI:** 10.1089/heq.2021.0177

**Published:** 2022-03-07

**Authors:** Charleigh J. Granade, Megan C. Lindley, Tara Jatlaoui, Amimah F. Asif, Nkenge Jones-Jack

**Affiliations:** ^1^ Immunization Services Division, National Center for Immunization and Respiratory Diseases, Centers for Disease Control and Prevention, Atlanta, Georgia, USA.; ^2^ Oak Ridge Institute for Science and Education, U.S. Department of Energy, Atlanta, Georgia, USA.

**Keywords:** adult immunization, vaccine, health equity, racial and ethnic disparities, health inequality

## Abstract

**Background:**

Adult vaccination coverage remains low in the United States, particularly among racial and ethnic minority populations.

**Objective:**

To conduct a comprehensive literature review of research studies assessing racial and ethnic disparities in adult vaccination.

**Search Methods:**

We conducted a search of PubMed, Cochrane Library, ClinicalTrials.gov, and reference lists of relevant articles.

**Selection Criteria:**

Research studies were eligible for inclusion if they met the following criteria: (1) study based in the United States, (2) evaluated receipt of routine immunizations in adult populations, (3) used within-study comparison of race/ethnic groups, and (4) eligible for at least one author-defined PICO (patient, intervention, comparison, and outcome) question.

**Data Collection and Analysis:**

Preliminary abstract review was conducted by two authors. Following complete abstraction of articles using a standardized template, abstraction notes and determinations were reviewed by all authors; disagreements regarding article inclusion/exclusion were resolved by majority rule. The Social Ecological Model framework was used to complete a narrative review of observational studies to summarize factors associated with disparities; a systematic review was used to evaluate eligible intervention studies.

**Results:**

Ninety-five studies were included in the final analysis and summarized qualitatively within two main topic areas: (1) factors associated with documented racial-ethnic disparities in adult vaccination and (2) interventions aimed to reduce disparities or to improve vaccination coverage among racial-ethnic minority groups. Of the 12 included intervention studies, only 3 studies provided direct evidence and were of Level II, fair quality; the remaining 9 studies met the criteria for indirect evidence (Level I or II, fair or poor quality).

**Conclusions:**

A considerable amount of observational research evaluating factors associated with racial and ethnic disparities in adult vaccination is available. However, intervention studies aimed at reducing these disparities are limited, are of poor quality, and insufficiently address known reasons for low vaccination uptake among racial and ethnic minority adults.

## Introduction

Immunizations are a safe and cost-effective strategy to reduce morbidity and mortality resulting from vaccine preventable illness. Despite the proven effectiveness of vaccination, adult immunization rates remain low in the United States. The Centers for Disease Control and Prevention (CDC) estimates that among U.S. adults, there are roughly 4,000 deaths attributable to invasive pneumococcal disease and between 3,000 and 49,000 deaths due to seasonal influenza annually.^1^ Racial and ethnic minority adults have higher hospitalization rates due to influenza and lower vaccination coverage than non-Hispanic White (White) adults.

Analysis of seasonal influenza data from 2009–2010 through 2018–2019 shows that the age-adjusted influenza hospitalization rates per 100,000 population among non-Hispanic Blacks (Black) is 68.1 and American Indian/Alaska Natives is 47.5 compared with 38.3 in White adults.^2,3^ Notably, Black (39.0%), Hispanic (37.5%), and individuals who identify as other or multiple race (41.4%) have persistently lower influenza vaccination coverage when compared with White (49.3%) adults.^4^

A diverse set of barriers contributes to low immunization uptake among the adult population, including lack of physician recommendations for vaccination, misconceptions about vaccination needs, lack of insurance coverage, and incomplete use of evidence-based strategies like standing orders or reminder-recall systems to increase routine adult vaccination.^5,6^ Suboptimal vaccination coverage among adults is further compounded by longstanding racial and ethnic disparities in vaccination uptake, reflecting stark health disparities and leading to a disproportionate burden of vaccine preventable diseases among several racial and ethnic minority groups.^7^

Social and health inequities, such as poverty and limited health care access, are multifaceted and interrelated issues that influence a wide range of health and quality-of-life outcomes.^8^ In addition, distrust of the health care system and history of discrimination in medical research among communities of color are barriers to those seeking health care when needed.^4^ It is important to understand and analyze these barriers and challenges to make progress toward overcoming health disparities in adult vaccination.

The purpose of this literature review is to highlight dynamic interactions at the individual, social, and environmental level, and summarize how they influence racial and ethnic disparities in adult immunization. The Social Ecological Model (SEM) is used as a guiding framework to classify factors associated with vaccination disparities. Also, intervention strategies to reduce racial and ethnic disparities in adult immunization are systematically evaluated.

## Methods

### Search strategy and selection criteria

We conducted a search in PubMed, Cochrane Library, and ClinicalTrials.gov using the following search string: (race OR ethnic* OR racial) AND (disparit* OR differen* OR gap) AND (vaccin* OR immuniz*) using the filter “Adult: 19+ years.” Studies in languages other than English were not included and publication year was restricted to 2000–2020. The search was conducted in June 2020 and returned 969 English-language abstracts; all articles were downloaded into EndNote X9.3.3 (Philadelphia, PA).

Preliminary abstract review was conducted by two authors (M.C.L. and C.J.G.), with full article reviews completed by at least one author to identify relevant studies for inclusion. Following complete abstraction of articles using a standardized template, abstraction notes and determinations were reviewed by all authors; disagreements regarding article inclusion/exclusion as well as SEM categorization were resolved by majority rule. The references of all included articles were reviewed to identify relevant articles not found in the initial literature search.

Of the 969 abstracts returned by our search, 758 were excluded after preliminary review for the following reasons: no abstract; duplicate abstract; epidemiological study (e.g., studies estimating prevalence, morbidity, and mortality); unlicensed (e.g., clinical trials of HIV vaccines) or nonroutine vaccines recommended for travel or limited at-risk groups (e.g., cholera, anthrax, smallpox, etc.); methodological study; no within-study comparison of different race/ethnic groups; not a study of differences in vaccine receipt (e.g., studies of vaccine-preventable disease, immunogenicity, or nonvaccination health behaviors); adults are not the subject of the study (e.g., studies of parents' willingness to vaccinate their children) or separate data on adults was not available; study not based in the United States; and study did not report original data (e.g., review articles and multiple publications of the same analysis). Many excluded abstracts fell into multiple exclusion categories.

The full text of 211 abstracts was obtained and assessed for eligibility. After review, 132 additional studies were excluded: 95 studies not evaluating factors associated with documented disparities, and 37 studies excluded for the reasons noted above. An additional three intervention studies were ineligible for the PICO (patient, intervention, comparison, and outcome) questions generated specifically for the intervention portion of the systemic review. Another 16 studies not captured by the original search were identified by the authors through manual bibliography review of all articles that met study inclusion criteria following full assessment.

**FIG. 1.**
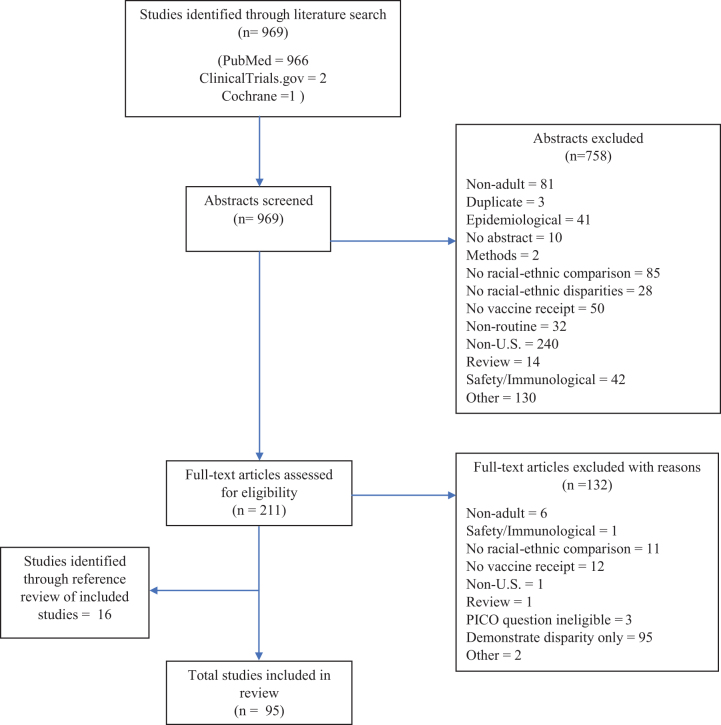
Flow chart of included and excluded observational and intervention studies.

As a result, 95 studies were included in the final analysis and summarized qualitatively within 2 main topic areas: (1) factors associated with documented racial-ethnic disparities in adult vaccination and (2) interventions aimed to reduce disparities or to improve vaccination coverage among racial-ethnic minority groups (Fig. 1).^9^

### Conceptual framework

The SEM, based on Brofenbrenner's ecological theory of development, which posits that behavior is influenced by multiple individual and environmental factors,^10–12^ is frequently used as a framework for prevention in health care.^13–15^ For this review, the authors applied an adjusted model that defines four interaction levels: individual, interpersonal, community, and environmental.^16^ The individual level comprises a person's attitudes, beliefs, and personal characteristics, while the interpersonal level is concerned with how the person interacts with others. The community level examines the settings and institutions in which the person interacts, and the environmental level looks at the high-level context of these interactions, including the policies that may shape behavior.

To organize literature review findings, articles focused on assessing factors associated with racial-ethnic disparities in adult vaccination were categorized using the four SEM levels above. Within each SEM level, articles are further grouped by themes regarding personal characteristics that influence vaccination decision making, interactions with health care and/or health care providers, characteristics of health care settings, and cultural or physical structural barriers to receiving vaccination services.

### Systematic review of intervention studies

We used the Preferred Reporting Items for Systematic Reviews and Meta-Analyses (PRISMA) guidelines to examine studies evaluating interventions to reduce adult vaccination disparities or improve vaccination uptake among racial-ethnic minority groups.^9^ Included studies assessed immunization of U.S. adult populations 19 years of age or older using either single-level or multilevel interventions for any adult vaccination recommended by CDCs Advisory Committee on Immunization Practices,^17^ as well as reported vaccination uptake or changes in uptake over time by race/ethnicity.

Articles were not restricted by study type (e.g., randomized controlled trials, cohort studies, or longitudinal studies), intervention type, population evaluated, or whether *a priori* intention to reduce racial and ethnic disparities in adult vaccination was evaluated.

Included articles were categorized into three intervention-specific PICO questions (Table 1). The PICO questions were designed with the aim of including racial-ethnic minority and nonminority participants eligible for vaccination and measuring disparities either through vaccination uptake or difference in vaccination uptake.

**Table 1.** Patient, intervention, comparison, outcome questions for systematic review of intervention studies

	Patient (P)	Intervention (I)	Comparison (C)	Outcome (O)	Time frame (T)
Direct/ideal study (PICO question 1)	Non-White and White adults in the United States eligible for vaccination	Provider/system/patient-specific intervention	No intervention	Difference in vaccination coverage/uptake by race/ethnicity	Before and after intervention
Indirect study (PICO question 2)	Non-White adults in the United States eligible for vaccination	Provider/system/patient-specific intervention	No intervention	Vaccination uptake	—
Indirect study (PICO question 3)	Non-White adults in the United States eligible for vaccination	Provider/system/patient-specific intervention	White adults in the United States eligible for vaccination	Vaccination uptake	—

Provider-specific interventions included health care provider recommendations; system-specific interventions; tracking and reminder system; community outreach activities; patient-specific interventions; health education; and awareness strategies.

PICO, patient, intervention, comparison, and outcome.

Two coauthors (T.C.J. and A.A.) independently reviewed each target article and abstracted the following information: authors, year of publication, funding source, study design and setting, timing, study population, vaccine of interest, intervention, comparison groups, outcomes, results, strengths and weaknesses of study, and level of evidence. To assess risk of bias, we independently graded each study according to the U.S. Preventive Services Task Force system to evaluate quality factors, including study design and methodological features such as assessment of potential confounders and outcome ascertainment.^18^ Due to the heterogeneity of study designs and reporting of outcomes, we did not analyze data quantitatively or compute summary measures.

## Results

### Reasons and associations

**FIG. 2.**
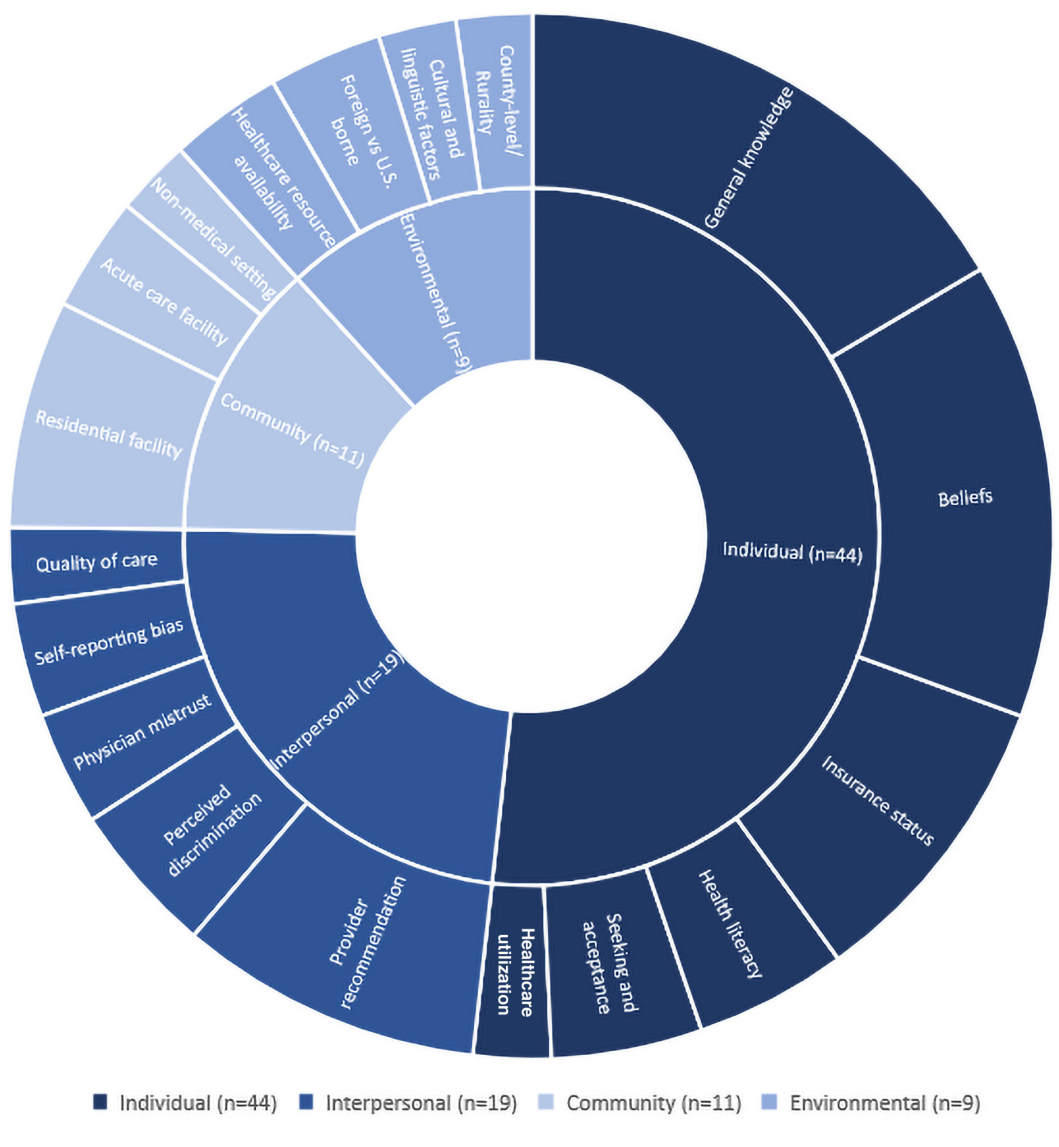
Reasons and associations for racial and ethnic disparities in adult vaccination by SEM level. SEM, Social Ecological Model.

The longstanding problem of racial-ethnic disparities in adult vaccination coverage is multifactorial. This section of the review discusses broad categories of factors that research suggests may be causally associated with differences in vaccination coverage by race/ethnicity, grouped by the SEM level at which they occur (Fig. 2).

### Individual

Some racial-ethnic minority individuals may have less knowledge about the need for vaccination or regarding vaccines in general compared with White individuals. Most studies evaluating knowledge focus primarily on differences in familiarity with Human papillomavirus (HPV) vaccination^19–25^; however, a minority also assess understanding the need for vaccination^26^ and vaccine recommendations.^27,28^

Studies have found both Hispanics and Blacks are less likely to be knowledgeable about HPV vaccine and/or disease,^29,30^ shingles vaccination,^31^ and influenza disease.^27,32^ Measured differences in general health literacy by race/ethnicity have also been associated with lower vaccine uptake among younger and older adults,^33–35^ although some studies find no effect of health literacy on vaccination disparities.^36^ Attitudes and beliefs regarding vaccination, including believing vaccines may cause harm, also contribute to observed racial-ethnic disparities in vaccination.^37–48^

Black adults have been shown to prefer social distancing, hand hygiene, or culturally specific home remedies in lieu of vaccination to prevent influenza.^44,46^ Differences in insurance status may mediate disparities in vaccination coverage, although available evidence is conflicting^14,49–55^ and racial-ethnic differences in vaccination persist among insured persons.^50^ Similarly, findings about how health care utilization affects vaccination disparities are mixed^56,57^; differences in utilization by race/ethnicity might reflect access barriers or other individual-level factors. All of these elements may contribute to lower vaccine-seeking behavior^58–60^ or vaccine acceptance^60,61^ by racial-ethnic minority adults compared with White adults.

### Interpersonal

Differences in receipt of provider recommendation for vaccination by race/ethnicity have been observed,^62–65^ yet accounting for these differences does not fully eliminate disparities.^66–68^ Provider recommendations can mitigate lower tendencies to initiate vaccination in racial-ethnic minority adults,^64,67^ although recommendations from a provider of the same race/ethnicity as the patient may carry more weight.^69^

The relationship of provider recommendations on vaccination uptake is further complicated by racial-ethnic differences in trust in providers. Studies suggest equal or greater trust by Hispanic relative to White patients,^70,71^ but lower trust by Black patients.^69,70^ Perceived discrimination among racial and ethnic minority groups is complex; research evaluating the impact of overt or subversive discrimination is limited.^72–75^

In one study, Black adults reporting health care stereotype threats from their provider were less likely to receive influenza vaccine,^72^ while another found racial consciousness and discrimination experienced within the health care setting were negatively associated with vaccine-seeking behavior.^74^

Potential differences in quality of care by patient race/ethnicity may be mitigated by greater contact with the health care system: disparities in influenza vaccination were lower among Hispanic adults reporting diabetes self-management education,^76^ and observed vaccination disparities among Medicare enrollees by race/ethnicity decreased with increasing numbers of health conditions.^77^ Some observed disparities may be accounted for by racial-ethnic differences in self-reporting vaccination, although findings are inconsistent regarding the direction of the bias.^78–80^

### Community

Ample evidence indicates the demographic composition of health care settings for both residential^81–86^ and acute^87–89^ care facilities substantially affects likelihood of vaccination. Studies consistently find patients in facilities serving higher proportions of racial-ethnic minority patients are less likely to be appropriately vaccinated^84–87^; such variability often compounds lower vaccination of minority patients within these facilities.^87^ The mechanism driving this association is unclear, although some studies postulate it is driven, in part, by differential use of vaccination promotion strategies such as standing orders.^83,89^

The availability of vaccination services in different settings in the community may also influence or widen disparities through use of nonmedical vaccination settings. Specifically, studies evaluating the use of pharmacies as alternative vaccination sites by racial-ethnic minority patients suggest Black adults have lower acceptance of receiving vaccinations within this setting compared with White patients, with findings for Hispanic adults conflicting.^90,91^

### Environmental

The context in which vaccination occurs, including cultural and linguistic factors, can influence vaccination disparities. Among Hispanic adults, Spanish-preferring adults are less likely than both White and English-preferring Hispanic adults to receive influenza^92,93^ and pneumococcal^92^ vaccinations. Notably, one study showed Spanish-preferring adults in longstanding Hispanic communities or areas of low linguistic isolation had the highest vaccination coverage of all three groups, suggesting the value of social support.^92^

Multiple studies also indicate lower knowledge about, interest in, and uptake of vaccination among foreign-born compared with U.S.-born racial-ethnic minority adults^94–96^; nativity may be a proxy for limited English proficiency or may signal cultural differences in perceived value of vaccination.

Findings regarding whether health care resource availability, which is associated with vaccination uptake, fully accounts for observed vaccination disparities between racial-ethnic minority and White adults are inconclusive.^97,98^ Racial-ethnic minority adults may be more affected than White adults by limited physician or vaccine supply,^98,99^ which has particular implications for racial-ethnic minority adults in rural areas.^98,100^

### Interventions

Our search strategy identified 12 intervention studies that addressed 3 intervention-specific PICO questions (Table 2). Three studies (Level II, fair quality) addressed the first PICO question and met inclusion criteria for direct evidence.^101–103^ The remaining nine studies answered the third PICO question meeting criteria for indirect evidence (Level I or II, fair or poor quality).^104–112^ None of the studies fulfilling the inclusion criteria for interventions addressed the second PICO question.

**Table 2.** Examining interventions assessing disparities by race and ethnicity for adult immunization

Author, year, funding source	Study design, location, timing	Population, vaccine, intervention	Comparison groups	Outcome, results	Strengths	Weaknesses	Level of evidence, quality
Daniels et al., 2006^107^ Cooperative Agreement of the American Association of Medical Colleges and the CDC; Robert Wood Johnson Minority Medical Faculty Development Award; National Institute on Aging, the National Institute of Nursing Research, and the National Center on Minority Health and Health Disparities, National Institutes of Health and the Department of Defense	Prospective cohort. Single-site, university-based general internal medicine clinic located in San Francisco, CA. June 1 to December 31, 2004.	All patients ≥18 years of age presenting to ED clinic and eligible for pneumococcal vaccination (*n*=370). Pneumococcal vaccine. Implemented nurse-initiated standardized offer of pneumococcal vaccination; those who declined were surveyed, given a strong physician recommendation, and offered vaccine.	Vaccinated vs. unvaccinated patients.	Compared to White patients, the adjusted OR (95% CI) for vaccine acceptance by race and ethnicity: Latino: adjusted OR 0.18 (0.03–1.03) African American: adjusted OR 0.34 (0.15–0.78) Russian: adjusted OR 0.26 (0.04–1.73) Asian: adjusted OR 0.71 (0.15–3.25) Other: adjusted OR 0.67 (0.11–4.04) Number (%) of patients accepting vaccination by race and ethnicity: White: 327 (88.0) Latino: 19 (83.0) African American: 71 (81.0) Russian: 23 (85.0) Asian: 57 (95.0) Other: 24 (89.0) Number (%) of patients declining vaccination by race and ethnicity: White: 43 (12.0) Latino: 12 (8.0) African American: 17 (19.0) Russian: 4 (15.0) Asian: 3 (5.0) Other: 3 (11.0)	Multiple race/ethnicity categories included in analysis. Real-life applicability and feasibility.	Single site. Small sample size. Not powered to detect difference by race categories. No baseline vaccination rate by race and ethnicity or comparison group without intervention during study period.	II-2, fair
Dexheimer et al., 2011^109^ Training grant from National Library of Medicine (LM T15 007450-03)	Prospective cohort. Single-site, adult ED in urban academic hospital located in Nashville, TN. January 31, 2006 to January 31, 2007.	Patients ≥65 years of age presenting to ED and eligible for vaccination (*n*=2062). Pneumococcal vaccine. Implemented computer-based reminder system, including EMR, computerized triage application, provider order entry system, and order tracking application.	White vs. Other race (as determined using ED billing system).	Total Pneumococcal vaccines administered: 222. White: 135 (60.1%) Other: 39 (17.6%) Compared to Whites, the adjusted odds of participants from a different racial or ethnic group receiving pneumococcal vaccine were 0.33 (0.24–0.45). Note: adjusted for sex, patient type, age, income, acuity, primary care physician, WR LOS, boarding LOS, occupancy of ED.	Real-life settings with all providers invited to participate. Analyzed outcome accounting for ED workload-related variables.	Single site. Unclear how many potential physicians could have participated. Standing orders not utilized. Race information only available for White or other categories; information not provided for eligibility sample. Study not powered to examine outcome by race.	II-2, poor
Hebert et al., 2010^104^ Source of funding not reported	Prospective cohort. Single-site, outpatient ambulatory facility of large, urban, safety-net hospital located in Miami, FL. September 2007 to January 2009.	Patients ≥18 years of age with systolic heart failure diagnosed by echocardiogram within 6 months of clinic presentation (*n*=549). Influenza and Pneumococcal vaccines. Implemented a disease management program inclusive of standardized vaccination protocol: (1) Assess influenza and pneumococcal vaccination status for every eligible patient. (2) Offer vaccination to all patients eligible at that visit. (3) Complete chart review after next visit (mean follow-up 2.8±2.9 months).	Percentage of patients vaccinated compared at baseline and at follow-up by race/ethnicity.	Adjusted odds (95% CI) of vaccination by race and ethnicity were 0.64 (0.37–1.05). Adjusted by age, race, sex, education, and various comorbidity factors. Vaccinated for influenza at baseline vs. follow-up: overall (28.3% vs. 50.4%) Black (34.2% vs. 53.0%) Hispanic (23.7% vs. 49.2%) Vaccinated for pneumococcal at baseline vs. follow-up: overall (30.7% vs. 65.5%) Black (30.7% vs. 63.9%) Hispanic (30.4% vs. 66.7%) At baseline, 22.0% of patients were vaccinated with both Influenza and Pneumococcal. Black and Hispanic patients separately had significantly higher influenza (*p*<0.01) and pneumococcal (*p*<0.01) vaccination rates at follow-up compared with baseline. No difference in vaccination rates for either vaccine observed between Black and Hispanic patients.	Real-life settings. Outcomes presented comparing race and ethnicity categories and at baseline vs. after intervention for each category.	Single site. Less generalizable population (disease state and predominately born outside the United States). Vaccination status at baseline obtained by self-report and medical record; proportion not reported.	II-2, poor
Humiston et al., 2011^108^ CDC National Immunization Program	Randomized, controlled trial. Six primary care centers in urban setting located in Rochester, NY. September 29, 2003 to January 22, 2004.	Patients ≥65 years of age with an active clinical record and resident of New York (*n*=3752). Influenza vaccine. Implemented the following activities: (1) Patient tracking to identify eligible patients and monitor vaccination status. (2) Provider reminders included in medical charts. (3) Mailed patient reminders. (4) Telephone outreach for patients without an appointment during 3-month flu vaccination period.	Standard practice of care vs. intervention.	Overall adjusted odds of vaccination for intervention: 6.27 (95% CI 5.41–7.22). Proportion of adults vaccinated by control vs. intervention group: African American: 25.0% vs. 60.0% White: 19.0% vs. 68.0% Hispanic: 25% vs. 51% Other: 20.0% vs. 70.0%	Large sample size. Powered to demonstrate >15% difference in vaccination rates. Groups similar at baseline by race and ethnicity, age, health insurance categories.	Described as randomized controlled trial; however, study did not follow randomized allocation and no concealment performed among patients and providers. Multivariate analysis not presented by covariates.	II-1, poor
Nowalk et al., 2006^101^ No source of funding reported	Prospective cohort. Two faith-based urban, low-income neighborhood health centers. August through October 2002.	Random sample of patients ≥50 years of age from each health center, with a recorded visit in the last year (*n*=375). Excluded patients with deafness, experiencing homelessness or with severe psychosis or dementia, residing in nursing home or outside Pittsburgh area. Pneumococcal vaccine. Implemented the following activities: (1) Patient- and provider-reminder systems. (2) Standing orders for vaccination. (3) Educational sessions for clinical staff. (4) No cost walk-in flu clinics.	Vaccinated compared with unvaccinated by age group and self-reported race.	Self-report of pneumococcal vaccination for Black (49.0%) and White (42.0%) participants (*p*=0.215). Pneumococcal vaccination among younger adults (50–65 years of age): Black: 39.0% White 26.0% *p*=0.061. Pneumococcal vaccination among older adults (65 years of age and older): Black 67.0% White 70.0% *p*=0.624.	Trained interviewers using computer-assisted telephone interviewing. Survey based on tested model, shown to be internally consistent and externally valid for influenza vaccination. Medical record considered gold standard for vaccine status.	Vaccinated compared with unvaccinated not provided by race and ethnicity. Baseline rates of pneumococcal vaccination assessed in a different study. Survey response rate 58%, unknown how respondents and nonrespondents differed.	II-2, fair
Nowalk et al., 2008^102^ Grant P01 HS10864 from AHRQ And P60 mD-000-207 from the NIH, National Center on Minority Health and Health Disparities for the EXPORT Health Project at the Center for Minority Health, University of Pittsburgh Graduate School of Public Health	Prospective cohort. Four inner-city health center intervention sites and fifth center serving as a control. Study timeline: 2000–2006 Pre-intervention: 2000–2001 Year 1: 2001/2 Year 2: 2002/3 Year 3: 2004/5 Year 4: 2005/6	Patients ≥50 years of age and with at least one visit in 2000 and 2005. Random sample of 150 eligible adults attending larger clinics and all eligible adults from smaller clinics (*n*=568). Influenza and Pneumococcal vaccines. Multilevel (culturally appropriate patient-, provider-, and system-oriented) interventions were selected by each clinic and implemented.	White patients were compared to Non-White patients, by site and study year.	The adjusted OR (95% CI) for Influenza vaccination was 1.06 (1.77–2.41) for non-White race. Following the intervention for the entire sample, the adjusted OR was 2.07 (1.77–2.41), adjusted for age, race, and sex. Comparison of influenza vaccination within the intervention group by study year (White vs. Non-White, respectively): Year 1: 49.2% vs. 49.6% Year 2: 41.3% vs. 43.3% Year 3: 41.6% vs. 46.6% Year 4: 48.6% vs. 49.1% The adjusted OR for Pneumococcal vaccination was 1.15 (0.66–1.98) for non-White race. Following the intervention for the entire sample, the adjusted OR was 1.21 (0.99–1.47) adjusted for age, race, and sex. Comparison of Pneumococcal vaccination within the intervention group by study year (White vs. Non-Whites, respectively): Year 1: 62.8% vs. 60.3% Year 2: 65.6% vs. 61.7% Year 3: 70.4% vs. 68.6% Year 4: 80.3% vs. 81.9%	Multicenter, multilevel intervention with control group assessed vaccination rates at baseline and each year of intervention. Multiple race and ethnicity categories included in analysis. Medical records used to assess vaccination status at baseline and during the study seasonal intervals and data abstracted by certified, independent researcher. Multivariate analysis conducted and controlled for age, race, and sex.	Various interventions at sites not reported or described. Non-White group not further described quantitatively by race and ethnicity categories. Intervention vs. nonintervention sites not described quantitatively by race and ethnicity. Smaller sample size for pneumococcal vaccine, likely not powered to detect difference between intervention and nonintervention groups.	II-2, fair
Plough et al., 2011^103^ No source of funding reported	Prospective cohort. Los Angeles County Department of Public Health H1N1 POD vaccination clinics. June 2009 through April 2010.	County residents meeting criteria for federally established priority groups for H1N1 vaccination. Stage 1: Dual delivery strategy developed to distribute 80% available vaccine to private health care sector, with remaining 20% distributed to residents through free PODs serving underinsured, low-income residents. Widespread public education campaigns. Stage 2: Vaccine Dispensing Stage 3: African American Outreach and Trust Building Partnership Strategy.	Estimates of the LA County population by race and ethnicity obtained from the Population Estimated Projections Systems.	Racial and ethnic distribution of residents vaccinated in PODs among estimated distribution of all residents in LA county: Black: 4965 (3.0%) among 946,994 (9.1%) Asian: 46,468 (28.5%) among 1,371,823 (13.2%) White: 33,434 (20.5%) among 3,123,783 (30.0%) Hispanic: 76,603 (47.0%) among 4,926,007 (47.3%) Native American: 537 (0.3%) among 26,837 (0.3%) Pacific Islander: 1080 (0.7%) among 23,251 (0.2%) With Whites as the reference group, observed the following rate ratios of POD vaccination by race and ethnicity: Black 0.5 Asian 3.2 White 1.0 Hispanic 1.5 Native American 1.9 Pacific Islander 4.3	Population-based vaccination data collected at the time of vaccination.	Intervention compared with LA County estimates, no comparison group that did not receive intervention.	II-2, fair
Stein and Nyamathi, 2010^111^ Nyamathi et al., 2009^112^ National Institute on Drug Abuse (Grants DA016147 and DA0107036)	Randomized, three-group, prospective, quasi-experimental design. Skid Row area of Los Angeles. September 2003 through August 2007.	Sheltered homeless (defined as homeless for at least 30 days) adults 18–65 years of age recruited from 12 homeless shelters, 4 residential drug treatment sites, outdoor locations with no history of HBV vaccination or HBV antibodies, and willing to undergo HAV/HBV/HCV/HIV testing at baseline and 6-month follow-up (*n*=865). Three-series Twinrix HAV/HBV vaccine. Participation in one of three programs: (1) NCMIT. (2) Standard hepatitis education, incentives, and tracking. (3) Standard hepatitis education and incentives only.	Outcome measures compared by intervention type and race.	Percent completion overall by race and ethnicity (*n*=530; 61.3%): African American: 71.9% White: 12.3% Latino: 13.2% Other 2.1% adjusted OR (95% CI) by race and ethnicity: African American: reference White, newly homeless: 0.41 (0.21–0.79) White, chronically homeless: 1.11 (0.65–1.88) Latino: 1.21 (0.77–1.90) Adjusted for intervention, age, sex, race/ethnicity, partner status, health status, recent self-help program: Arm 1: 68.0% Arm 2: 61.0% Arm 3: 54.0% adjusted OR (95% CI) by intervention program: Group 3: reference Group 1: 1.85 (1.13–3.04) Group 2: 1.51 (0.93–2.44) The 3 risk factors (needle sharing, sexual history, and history of incarceration) explained 2% of the variance in completion and 1% of the variance in loss. Adding the other variables increased the variance explained to 14.0% for completion and 13.0% for loss.	Instruments used for data collection tested and validated among homeless populations, administered by face-to-face interview. Intent-to-treat analyses. Iterative and more comprehensive predictive models used to evaluate completion and attrition in NCMIT group using known risk factors for HBV and demographic variables.	Groups differed at baseline by race and ethnicity. Results not presented by arm and race and ethnicity.	II-2, fair
Schensul et al., 2009^105^ Source of funding not reported	Randomized controlled trial of multilevel intervention. Two public senior housing buildings in Hartford, CT. Conducted from 2004 through 2006.	Low-income, ethnically diverse seniors ≥62 years of age, with smaller resident population of younger disabled adults (younger than 62 years). Influenza vaccine. There were three levels of intervention: (1) Create regional advisory group to provide support and advocate for flu activities and access. (2) Engage building management to support flu vaccination and other public health activities. (3) Organize and empower residents as peer health advocates to promote building-wide provaccination culture and practices.	Intervention vs. control building residents' rates of vaccination (baseline and post-intervention).	The self-reported vaccination rate increased from 30.4% to 71.0% of respondents in the intervention building. A test of difference between proportions showed a significant difference between the increase in vaccination in the control building (18.0%) and the intervention building (41.0%) (*p*=0.010). The effect was greater for Puerto Ricans (*p*=0.002, change in odds from 2.35 to 6.83) than for Blacks (nonsignificant, confirmed with logit linear analysis).	Well defined multilevel intervention. Resident groups in intervention and control building similar at baseline by age, education, and income. Multiyear study able to assess change in flu vaccination coverage. Campaign efforts and intervention tailored with VIP Resident Committee input. Sustainability built into intervention planning.	Small sample size. Flu vaccination coverage at baseline and post-intervention for both groups by self-report. Outcomes not presented comparing race and ethnicity categories and at baseline vs. after intervention for each category.	I, fair
Schwartz et al., 2006^110^ AAMC/CDC Cooperative Grant U36/CCu319276 CFDA 93.283	Prospective cohort study. Seven primary care practices and members of MetroNet, the metropolitan Detroit practice-based research network October 2003 through January 2004.	Patients ≥65 years of age at one of the participating MetroNet offices (*n*=454). Excluded patients who had already received the current vaccine and those who stated vaccination as a reason for visit. Influenza vaccination. Nonphysician-initiated standardized offer of influenza vaccination.	Proportion of African American, White, and other race/ethnicity patients regarding vaccine acceptance.	Adjusted OR (95% CI) of vaccine acceptance African American vs. White 1.20 (0.63–2.29); *p*=0.57. Percentage of vaccine acceptance by race (*p*=0.26): Black: 136/181 (75.6%) White: 191/236 (68.9%) History of previous vaccination was the only statistically significant predictor of vaccine acceptance.	Powered to demonstrate >15% difference in vaccination rate between White and African American participants. Intervention clearly defined with none of the participating offices having standing orders policy before intervention. Multiple race and ethnicity categories included in analysis. Vaccination status by medical record review.	Self-report used to record baseline vaccination status. Racial and ethnic groups differed at baseline in age and educational status. No comparison group that did not receive intervention.	II-2, poor
Winston et al., 2007^106^ Contract 0000HCJ4-s004-07797 from the CDC	Randomized controlled trial. Five managed care network general medicine clinics in Atlanta, GA, March 2004 through March 2005.	Unvaccinated patients ≥18 years of age with diabetes mellitus, chronic heart failure, or coronary artery disease (*n*=3711). Unvaccinated elderly patients ≥65 years of age and participating in Medicare (*n*=2395). Pneumococcal vaccine. Mailed reminders to intervention group participants before scheduled clinic visits, followed by a telephone recommendation by a trained nurse.	Outcome measures compared by study arm and race.	Proportion vaccinated among those in chronic disease group reached by telephone intervention (*n*=1845): Non-Hispanic Black: 25.0% Non-Hispanic White: 34.0% *p*=0.03 Proportion vaccinated among those in elderly group reached by telephone intervention (*n*=1198): Non-Hispanic Black: 24.0% Non-Hispanic White: 34.0% *p*=0.03	Randomization and blinding done. Clear definition of intervention. Large overall sample size.	Vaccination status assessed by administrative database and may have not captured vaccination outside of clinic either at free event or paid out of pocket. Results by race/ethnicity, collected by self-report, were limited to intervention patients only. In the analyses model, race and ethnicity were not included as a direct measure, but using clinic group as a proxy.	II-2, poor

AHRQ, Agency for Healthcare Research and Quality; CDC, Centers for Disease Control and Prevention; CI, confidence interval; ED, emergency department; EMR, electronic medical record; HAV, hepatitis A virus; HBV, hepatitis B virus; HCV, hepatitis C virus; HIV, human immunodeficiency virus; LOS, length of stay; NCMIT, Nurse Case Management with Incentives and Tracking; NIH, National Institutes of Health; OR, odds ratio; POD, point of dispensing; WR, waiting room.

Three prospective studies provided direct evidence and examined the impact of multilevel interventions on adult vaccination disparities in pneumococcal and/or influenza vaccination coverage.^101–103^ Each of the three studies examined adult vaccination rates before and after implementing multilevel interventions using a comparison group; two of these studies demonstrated the interventions improved pneumococcal and/or influenza vaccination rates among racial-ethnic minority groups when compared with the White adult group.^101,102^ Details regarding the multilevel interventions implemented and evaluated in each of these two studies were not further described.

The third study demonstrated effectiveness of the intervention that comprised countywide large-scale vaccine distribution and public education activities for H1N1 vaccination in White and racial-ethnic minority participants, but its effect on vaccination disparities was not examined.^103^ Therefore, the authors concluded that the increase in vaccination rates was not commensurate with their respective population estimates, highlighting any improvement was inadequate.

Of the nine studies that examined adult vaccination rates after, and not before, an intervention by race/ethnicity, three implemented multilevel interventions,^105,111,112^ two implemented provider-specific interventions,^107,110^ and four studies implemented system-specific interventions.^104,106,108,109^ All three studies implementing multilevel interventions observed a significant increase in vaccination rates among racial and ethnic minority groups. However, the effect of these interventions varied by race/ethnicity, with multilevel interventions more effective for increasing influenza vaccination in Puerto Rican adults (no comparison to White adults reported)^105^ and hepatitis A and B vaccination series completion in unhoused Black adult populations compared with Whites.^111,112^

Similarly, a study focusing on provider-specific interventions demonstrated that Black race was a significant predictor of pneumococcal vaccine refusal when compared with Asians and Whites^107^; however, a similar study showed no difference in odds of influenza vaccine acceptance among White and Black populations.^110^ The remaining four studies utilizing system-specific interventions, including reminder-recall and standardized vaccination protocols, did not demonstrate increases in the likelihood of getting vaccinated or the overall vaccination rates among racial and ethnic minorities.^104,106,108,109^

The strengths of the fair quality studies providing direct evidence and that address PICO question 1 include the following: conducted at multiple sites, implemented multilevel interventions in different racial and ethnic minority groups, and analyzed and reported results for each minority group. These studies also used medical records to assess vaccination status at baseline and post-intervention.

Notably, some studies were not designed *a priori* to evaluate racial disparities in vaccination and did not clearly describe the multilevel interventions implemented. Furthermore, indirect studies that answered PICO question 3 were found to be poor quality evidence, with included interventions conducted at a single site and used self-report to document vaccination status. Overall included studies evaluating interventions to reduce racial and ethnic minority disparities in adult vaccination are insufficient and do not adequately address system-specific or provider-specific interventions.

## Discussion

A considerable amount of research regarding racial and ethnic disparities in adult vaccination has been published over the last two decades, with an overwhelmingly large proportion of studies focused on observational findings and a more limited set concentrated on addressing observed disparities. Many studies highlight individual-level factors associated with vaccination disparities; however, factors at the interpersonal, community, and environmental levels are simultaneously present and likely contribute to observed differences. The limited research evaluating interventions aimed at addressing these observed disparities further underscores the lack of progress in identifying and implementing actionable strategies to advance health equity in adult immunization.

Determinants of racial and ethnic disparities in adult vaccination are multifactorial; however, most available research is predominantly centered on factors that occur at the individual level. Application of the SEM in prevention research is widely accepted; a systematic review of multilevel prevention models for vaccination and screening found application of this model to be effective in determining vaccination behaviors when a multisystem approach was used.^113^

Similarly, a comprehensive study of influenza vaccination uptake concluded variables at the individual, interpersonal, and institutional levels had the greatest impact on influenza vaccination uptake.^14^ In this review, we identified numerous studies that suggest characteristics at every SEM level are associated with low vaccination coverage in racial and ethnic minority populations. Although actions to increase vaccine confidence and coverage are currently being implemented at the federal level, additional stakeholder efforts that address barriers at multiple levels of the SEM are needed to make any tangible progress in addressing these disparities.

Multilevel interventions addressing multiple SEM levels have been shown to be effective in reducing adult vaccination disparities.^114^ In this review, most of the included intervention studies with fair quality evidence demonstrated effectiveness in increasing vaccination rates among racial and ethnic minority groups through use of multilevel interventions. These multilevel interventions include strategies that improve vaccine demand through patient education activities and health care provider interactions, and ensure equitable vaccine access in health care settings.

The Community Preventive Services Taskforce also recommends implementing two or more interventions that focus on increasing community demand and/or access to vaccinations to improve vaccination rates in targeted populations and reduce disparities.^115^

However, only a limited number of included studies implemented provider-level interventions focusing on health care provider recommendations and hospital-based strategies. In addition, very few studies evaluated community- or environmental-level strategies such as use of extended clinical hours^116^ or pharmacy-based initiatives.^117^ These findings underscore the need for additional research to evaluate interventions that address known factors associated with immunization disparities in racial and ethnic minority groups to develop meaningful strategies for increasing vaccine equity across various traditional and nontraditional health care settings.

One of the primary factors identified by this review as driving racial and ethnic vaccination disparities at the individual level was patient attitudes toward vaccination, in particular, the belief that vaccines are harmful. The higher prevalence of these attitudes in racial and ethnic minority adults relative to White adults could be due to differences in risk tolerance by race or ethnicity, such that avoiding the relatively remote chance of a serious adverse event following immunization is valued more highly than the projected benefits of vaccination.

Mistrust of health care providers and conventional medicine in general may influence these attitudes: studies suggest medical mistrust is higher among racial and ethnic minority adults, particularly Black adults, and higher medical mistrust is associated with reduced intent to receive vaccines.^118,119^ However, medical mistrust is an incomplete explanation that ignores perceptions and experiences of racism in medical settings^120–122^; research suggests that physician communication with Black adults may be less patient centered or engaged.^123^ Addressing health care provider communication with racial and ethnic minority patients, as well as increasing racial concordance and medical workforce diversity,^124^ may reduce vaccine hesitancy in these populations.

This literature review has several limitations. First, the literature search string was not inclusive of additional health equity terms such as “inequities” or “inequalities,” which may have introduced study imprecision. Although the authors conducted a manual bibliography review of all articles meeting inclusion criteria, omission of these terms may have resulted in incomplete capture of available research. Second, assessment of within-population heterogeneity as factor impacting vaccination disparities was not possible due to most included studies using broad race/ethnicity definitions. Finally, the themes described in each SEM level are reflective of available research only; therefore, additional interactions relevant to vaccine decision making and practices among racial and ethnic populations are not discussed.

## Conclusions

This literature review presents a comprehensive examination of published racial and ethnic disparity research in adult vaccination over the past 20 years. Although we identified few studies focused on how to decrease racial and ethnic disparities in adult vaccination, our findings suggest broader use of multilevel interventions addressing patient concerns, as well as structural and contextual barriers to access, may help increase routine vaccination uptake in communities of color. Implementation of this type of intervention at the scale needed to evaluate and demonstrate change will likely require dedicated resources and focus from federal, state, and local public health officials, health care providers, and community partners.

Importantly, such activities should be guided by early and ongoing investment from the affected community to maximize the benefits of the intervention.^125^ Only through these concerted efforts and partnerships can tangible advancement in reducing longstanding adult vaccination inequities resulting from a national legacy of structural racism be accomplished.

## Abbreviations Used

AHRQ: Agency for Healthcare Research and Quality
CDC: Centers for Disease Control and Prevention
CI: confidence interval
ED: emergency department
EMR: electronic medical record
HAV: hepatitis A virus
HBV: hepatitis B virus
HCV: hepatitis C virus
HIV: human immunodeficiency virus
HPV: human papillomavirus
LOS: length of stay
NCMIT: Nurse Case Management with Incentives and Tracking
NIH: National Institutes of Health
OR: odds ratio
PICO: patient, intervention, comparison, outcome
POD: point of dispensing
PRISMA: Preferred Reporting Items for Systematic Reviews and Meta-Analyses
SEM: Social Ecological Model
WR: waiting room
